# Study protocol: E-freeze - freezing of embryos in assisted conception: a randomised controlled trial evaluating the clinical and cost effectiveness of a policy of freezing embryos followed by thawed frozen embryo transfer compared with a policy of fresh embryo transfer, in women undergoing in vitro fertilisation

**DOI:** 10.1186/s12978-019-0737-2

**Published:** 2019-06-13

**Authors:** Abha Maheshwari, Siladitya Bhattacharya, Ursula Bowler, Daniel Brison, Tim Child, Christina Cole, Arri Coomarasamy, Rachel Cutting, Stephen Harbottle, Pollyanna Hardy, Edmund Juszczak, Yacoub Khalaf, Jennifer J. Kurinczuk, Stuart Lavery, Clare Lewis-Jones, Nick Macklon, Nick J. Raine-Fenning, Madhurima Rajkohwa, Graham Scotland, Stephen Troup

**Affiliations:** 1 University of Aberdeen, Aberdeen, UK; 2 University of Cardiff, Cardiff, UK; 3 University of Oxford, Oxford, UK; 4 St. Mary’s Hospital, Manchester, UK; 5 Birmingham Women’s Hospital, Birmingham, UK; 6 Jessop Wing Maternity Unit, Sheffield, UK; 7 IVF Cambridge, Cambridge, UK; 8 University of Birmingham, Birmingham, UK; 9 Guy’s and St. Thomas’ Hospital, London, UK; 10 IVF Hammersmith, London, UK; 11 Formerly Fertility Network, London, UK; 12 London Women’s Clinic Group, London, UK; 13 University of Nottingham, Nottingham, UK; 14 Care Fertility, Birmingham, UK; 15 Reproductive Health, Manchester, UK

**Keywords:** IVF, Fertility, Frozen thawed embryo transfer, Fresh embryo transfer, OHSS, Elective freezing, Assisted conception, Receptivity

## Abstract

**Background:**

Infertility affects one in seven couples; many of these need in vitro fertilisation (IVF). IVF involves external hormones to stimulate a woman’s ovaries to produce eggs which are harvested surgically. Embryos, created in the laboratory by mixing eggs with sperm, are grown in culture for a few days before being replaced within the uterus (fresh embryo transfer). Spare embryos are usually frozen with a view to transfer at a later point in time – especially if the initial fresh transfer does not result in a pregnancy. Despite improvements in technology, IVF success rates remain low with an overall live birth rate of 25–30% per treatment. Additionally, there are concerns about health outcomes for mothers and babies conceived through IVF, particularly after fresh embryo transfer, including maternal ovarian hyperstimulation syndrome (OHSS) and preterm delivery. It is believed that high levels of hormones during ovarian stimulation could create a relatively hostile environment for embryo implantation whilst increasing the risk of OHSS. It has been suggested that freezing all embryos with the intention of thawing and replacing them within the uterus at a later stage (thawed frozen embryo transfer) instead of fresh embryo transfer, may lead to improved pregnancy rates and fewer complications. We aim to compare the clinical and cost effectiveness of fresh and thawed frozen embryo transfer, with the primary aim of identifying any difference in the chance of having a healthy baby.

**Methods:**

E-Freeze is a pragmatic, multicentre two-arm parallel group randomised controlled trial where women aged ≥18 and < 42 years, with at least three good quality embryos are randomly allocated to receive either a fresh or thawed frozen embryo transfer. The primary outcome is a healthy baby, defined as a term, singleton, live birth with appropriate weight for gestation. Cost effectiveness will be calculated from a healthcare and societal perspective.

**Discussion:**

E-Freeze will determine the relative benefits of fresh and thawed frozen embryo transfer in terms of improving the chance of having a healthy baby. The results of this pragmatic study have the potential to be directly transferred to clinical practice.

**Trial registration:**

ISRCTN registry: ISRCTN61225414. Date assigned 29/12/2015.

## Plain English summary

Infertility is common, affecting one in seven couples in the UK, many of whom will ultimately need in vitro fertilisation (IVF). IVF involves several steps; initially hormones are used to stimulate the woman’s ovaries to produce eggs, which are then removed through a surgical procedure. Next, embryos are created in the laboratory by mixing eggs with sperm. In conventional IVF, these embryos are grown for a few days before the best available embryo is placed inside the uterus in a process known as fresh embryo transfer. Any spare embryos are then usually frozen.

Despite improvements in technology, IVF success rates remain low and there are associated risks to women and babies. It is thought that the high levels of hormones used to stimulate a woman’s ovaries to produce eggs could create a relatively hostile environment for the embryo within the uterus. Freezing embryos and placing them in the uterus at a later stage may lead to improved pregnancy rates and fewer complications. This study aims to compare fresh and thawed frozen embryo transfer, with the main aim of identifying any differences in the chances of having a healthy baby as well as differences in costs. The study will be available to couples at selected fertility centres across the UK.

## Background

The National Institute for Health and Care Excellence (NICE) recommends IVF as the definitive treatment for prolonged unresolved infertility [1]. IVF involves several steps. Initially, hormones are used to stimulate a woman’s ovaries to produce eggs which are harvested surgically. Next, embryos are created in the laboratory by mixing eggs with sperm. In conventional IVF these are grown in culture for a few days before being replaced within the uterus by a process known as fresh embryo transfer. Spare embryos are usually frozen with a view to transfer at a later point in time – especially if the initial fresh transfer does not result in a pregnancy. Despite improvements in technology, IVF success rates remain low with an overall live birth rate of 25% per treatment. Additionally, there are concerns about health outcomes for mothers and babies conceived through IVF, particularly after fresh embryo transfer, including maternal ovarian hyperstimulation syndrome (OHSS) and perinatal morbidity.

A possible cause for sub-optimal live birth rates as well as adverse maternal and perinatal outcomes following IVF is the impact of exogenous hormones used for ovarian stimulation on the lining of the uterine cavity. High levels of oestrogen produced by the ovary in response to this treatment affect uterine receptivity, reducing the chances of successful implantation and placentation. It has been suggested that avoiding embryo transfer at a time when the uterus is less receptive could improve success rates. Such a strategy also reduces the risk of OHSS by ensuring that a pregnancy does not occur in the presence of hyperstimulated ovaries.

Preliminary data from small randomised trials from Iran [2] and the USA [3, 4] suggest that a strategy of not replacing embryos when they are created but freezing them followed by thawed frozen embryo transfer into the uterus at a later date improves pregnancy rates. A meta-analysis of data from these three RCTs [5] has shown higher pregnancy rates following thawed frozen embryo transfer (odds ratio 1.32, 95% CI 1.10 to 1.59).

However, these existing trials have a number of significant limitations:

- They reported implausibly high pregnancy rates (e.g. 84% per embryo transfer), which are far in excess of those reported by national and international registries.
- Key outcomes including healthy baby, live birth, costs, safety and acceptability were not measured by any of the trials.
- They were limited in terms of design with highly selected populations, inadequate sample sizes and per protocol analysis rather than by intention-to-treat and conduct, since all involved co-interventions which were not accounted for in the analysis.

One of the publications [2] has been retracted on the grounds of serious methodological flaws. Hence, the current evidence base comprising two small trials of suboptimal quality is not sufficiently robust to support a radical change in clinical practice. Additionally, their results cannot be directly applied to a UK setting due to very different regulatory and funding arrangements. There is, therefore, an urgent need to perform a definitive randomised controlled trial in the UK evaluating elective freezing of embryos followed by subsequent thawed frozen embryo transfer in terms of clinical and cost effectiveness.

A two-arm parallel group randomised controlled trial is proposed across multiple fertility centres in the UK. Women ≥18 and < 42 years of age undergoing their first, second or third IVF/ICSI treatment, with at least 3 good quality embryos will be randomised to either fresh embryo transfer (standard treatment arm) or thawed frozen embryo transfer, this typically will take place after 4 to 6 weeks and always within 3 months of egg retrieval (intervention treatment arm).

A single episode of thawed frozen embryo transfer (after elective freezing of embryos) will be compared to a single episode of fresh embryo transfer with a healthy baby (defined as a live singleton baby born at term with an appropriate weight for gestation) – the primary outcome.

With 90% power and a two-sided 5% level of statistical significance, we will need to randomise 1086 couples (543 in each arm) to show an absolute difference in the primary outcome of at least 9% (e.g. from 25 to 34%), between fresh and thawed frozen embryo transfer respectively.

A full economic evaluation will assess the costs and consequences of the new strategy compared with standard practice. The trial data will be combined with modelling to estimate the long-term costs of health and social care using a previously developed decision analytic model.

## Methods/ design

**Fig. 1:**
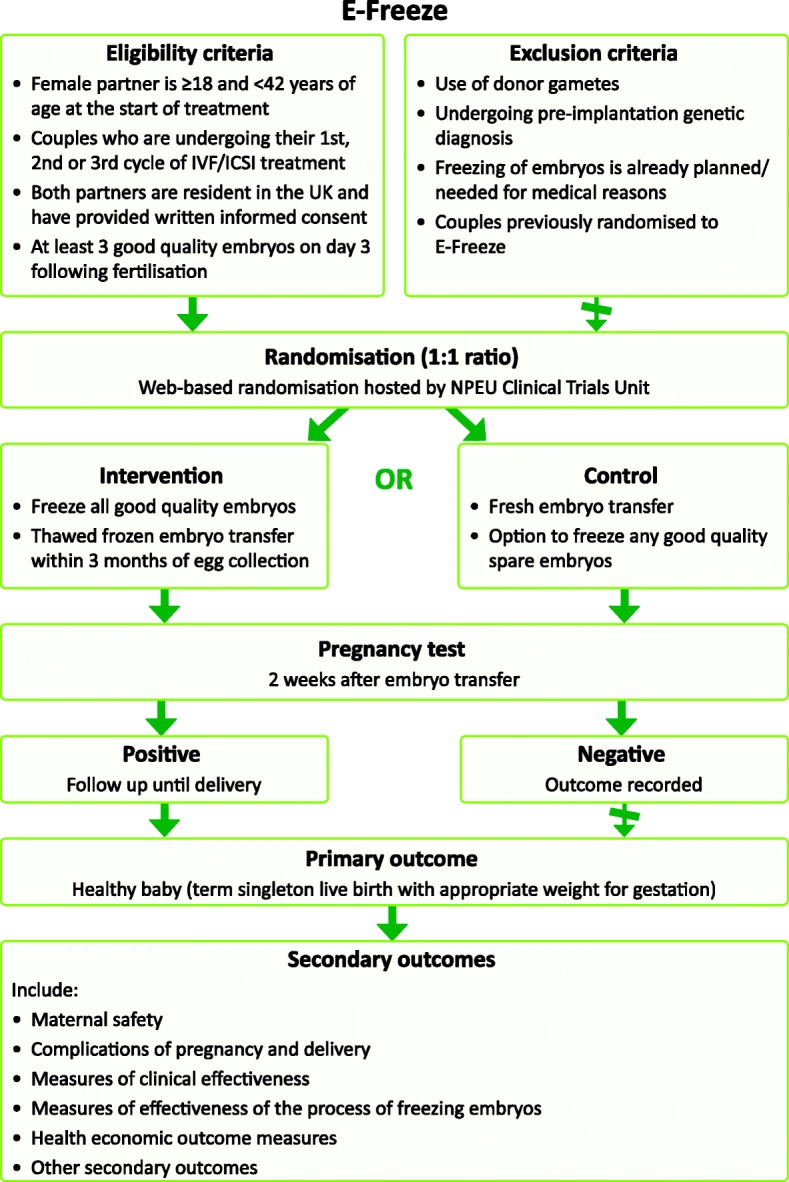
E-Freeze Trial Design

A pragmatic multi-centre two-arm parallel group randomised controlled trial to evaluate the effectiveness of a policy of freezing embryos followed by thawed frozen embryo transfer with a policy of fresh embryo transfer in women undergoing in vitro fertilisation will be conducted. All clinical elements of IVF treatment, apart from the randomised interventions, will be carried out according to local protocols. The trial design is summarised in Fig. 1.

### Number of participants

1086 couples undergoing their first, second or third cycle of IVF/ICSI treatment will be recruited from fertility centres across the UK.

### Inclusion criteria

- The female partner is ≥18 and < 42 years of age at the start of treatment (i.e. start of ovarian stimulation)
- Couples who are undergoing their first, second or third cycle of IVF/ICSI treatment, where a cycle is defined as egg collection following ovarian stimulation.
- Both partners are resident in the UK
- Both partners are able to provide written informed consent
- At least 3 good quality embryos (as defined by the Association of Clinical Embryologists, UK) on day 3 after egg collection (day of egg collection is counted as day 0). Good quality embryos on day 3 are defined as those with 6–8 cells grade 3/3 or above using the agreed national grading scheme [6].

### Exclusion criteria

- Couples using donor gametes
- Pre-implantation genetic testing is being planned
- Elective freezing of all embryos is planned for medical reasons (e.g. severe risk of OHSS)
- Couples previously randomised to E-Freeze

### Primary outcome

The primary outcome is a healthy baby. A healthy baby is defined as a live singleton baby born at term (between 37 and 42 completed weeks of gestation) with an appropriate weight for gestation (weight between 10th and 90th centile for that gestation based on standardised charts).

### Secondary outcomes

The secondary outcomes relate to maternal safety, complications of pregnancy and delivery, measures of clinical effectiveness, measures of effectiveness of the process of freezing embryos and health economic outcome measures.

### Maternal safety outcome

Ovarian hyperstimulation syndrome (OHSS) – defined and classified as per the Royal College of Obstetricians and Gynaecologists’ Green-top Guideline [7].

### Complications of pregnancy and delivery outcomes

- Vanishing twin or triplet (defined as either: more early fetal hearts detected than babies born, more gestational sacs than babies born or more gestational sacs than fetal hearts detected)
- Miscarriage rate (defined as pregnancy loss prior to 24 weeks of gestation)
- Ectopic pregnancy
- Termination
- Gestational diabetes mellitus (GDM)
- Multiple pregnancy (defined as more than one fetal heart or more than one gestational sac detected)
- Multiple births (including live and stillbirths)
- Hypertensive disorders of pregnancy (essential hypertension, pregnancy induced hypertension, pre-eclampsia and eclampsia)
- Most severe hypertensive disorder (from least to worst: essential hypertension, pregnancy induced hypertension, pre-eclampsia and eclampsia)
- Antepartum haemorrhage (any bleeding per vaginum after 28 weeks of pregnancy including placenta praevia and placental abruption)
- Onset of labour (spontaneous, induced or planned caesarean section)
- Mode of delivery for each baby (normal vaginal delivery, instrumental vaginal delivery or caesarean section)
- Preterm delivery (defined as delivery at < 37 completed weeks)
- Very preterm delivery (defined as delivery at < 32 completed weeks)
- Low birth weight (defined as weight < 2500 g at birth)
- Very low birth weight (defined as weight < 1500 g at birth)
- High birth weight (defined as weight > 4000 g at birth)
- Large for gestational age (defined as birth weight > 90th centile for gestational age at delivery, based on standardised charts)
- Small for gestational age (defined as birth weight < 10th centile for gestational age at delivery, based on standardised charts)
- Congenital anomaly/birth defect (all congenital anomalies/birth defects identified will be included)
- Perinatal mortality (stillbirth or late as well as early neonatal deaths, up to 28 days after birth)

### Measures of clinical effectiveness outcomes

- Live birth rate (this is a live birth episode, i.e. twins will count as one)
- Singleton live birth rate
- Singleton live birth rate at term
- Singleton baby with appropriate weight for gestation
- Pregnancy rate (defined as positive pregnancy test at two weeks +/− three days after embryo transfer)
- Clinical pregnancy rate (defined as the presence of at least one fetal heartbeat at ultrasound between six and eight weeks of gestation; ectopic pregnancy counts as a clinical pregnancy; multiple gestational sacs count as one clinical pregnancy)

### Measures of the effectiveness of the process of freezing embryos outcomes

- Total number of embryos frozen, thawed and transferred for all randomised couples
- Proportion of thawed embryos that were then transferred for all randomised couples
- Failure of all embryos to survive after thawing leading to no embryo transfer

### Health economic outcome measures

- Cost to the health service of treatment, pregnancy and delivery care
- Modelled long-term costs of health and social care, and broader societal costs

### Other secondary outcomes

- Evaluation of emotional state (for both the female and male partners) when they are advised of delay in embryo transfer in frozen arm

### Study description

All couples embarking on their first, second or third cycle of IVF/ICSI or a combination of both, will receive a letter of invitation introducing the trial and a copy of the Participant Information Leaflet. This will be sent with their clinic appointment. Participant Information Leaflets will also be distributed to couples attending an introductory patient information session, which will occur before their first clinic appointment. Eligible couples will be invited by a clinician involved in their care to participate in the trial. They will have the opportunity to speak to a research nurse to ask questions.

Consent forms need to be signed by both partners separately. This can be done at their clinic appointment or at a subsequent visit up until but prior to the procedure of egg collection, by an appropriately delegated member of the team. After consent, couples will each fill in a short questionnaire on how they are feeling emotionally. Each participant will seal their questionnaire in an envelope after completion and questionnaires will be destroyed unopened if the couple do not proceed to randomisation. Data needed for randomisation/minimisation will be recorded by the consent and randomisation program. On the first day after egg collection the embryologist or research delegate will confirm consent during a routine phone call to the couple to discuss the outcome of fertilisation.

On the third day after egg collection couples with at least three good quality embryos will be randomised by the embryologist or research nurse to fresh or thawed frozen embryo transfer. Good quality embryos on day three are defined as those with 6–8 cells grade 3/3 or above using the agreed national grading scheme [6]. Couples will be informed of their randomisation allocation by the embryologist or research delegate during their routine phone call on day three.

Couples who are randomised to thawed frozen embryo transfer will be contacted by the research nurse or research delegate within three working days post-randomisation to plan thawed frozen replacement treatment typically four to six weeks later and usually within three months of egg collection.

At embryo transfer (cleavage or blastocyst for fresh, or typically four to six weeks later for thawed frozen embryo transfer but usually within three months) couples will be asked to complete a short questionnaire to assess additional costs related to the treatment, and to repeat the emotions questionnaire they filled in at consent.

Women who have a positive pregnancy test two weeks (+/− three days) after embryo transfer will be contacted by their research nurse (by telephone) to record pregnancy events and outcomes at: 12 and 28 weeks of gestation and again around six weeks after delivery. Outcomes presenting themselves >six weeks post-delivery will not be recorded. All women who conceive by IVF/ICSI are followed up by their IVF centres routinely, as there is a mandatory requirement to report early pregnancy outcomes as well as delivery outcomes to the regulatory body, the Human Fertilisation Embryology Authority (HFEA), including stillbirth, congenital anomalies and perinatal mortality. Usually this information is provided to each IVF clinic by couples themselves. Alternatively, clinic staff contact couples by telephone to collect this information to report it back to the HFEA.

**Fig. 2:**
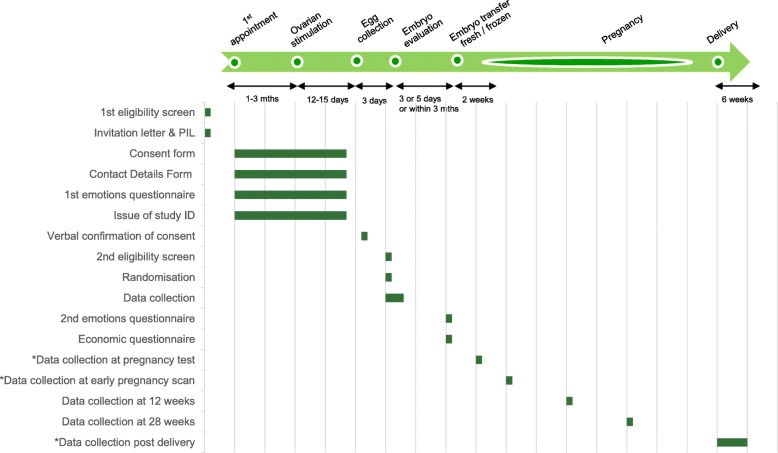
Study Matrix

The participant pathway and data collection points are shown in the Study Matrix (Fig. 2).

### Ineligible and non-recruited participants

Details of all consenting couples will be entered on a dedicated secure online database. It is anticipated that a proportion of those consented may not proceed to randomisation; the reasons for this will be recorded (if available) including non-availability of three good quality embryos on day three. As part of routine practice, the embryologist contacts the couple by telephone to let them know how many eggs are fertilised (next day after egg collection, day one) and the quality of their embryos (on day three after egg collection). The embryologist or research delegate will confirm consent on day one and inform them whether or not they fulfilled the final inclusion criteria (at least three good quality embryos on day three) and which arm they have been randomised to at the time of their routine phone call on day three. The research nurse will then contact the couple if they have not fulfilled the inclusion criteria to answer any queries and offer follow-up in the clinic. Couples not proceeding to randomisation will be offered the most appropriate standard treatment. All clinics have access to supportive counselling as a mandatory requirement of the regulatory authority.

### Randomisation

Randomisation will be performed after the creation of embryos, three days post egg collection. This will minimise the randomisation-to-intervention time interval as embryos are either transferred at the cleavage or blastocyst stage. Once all eligibility criteria are established (including ensuring that three or more good quality embryos are available), women will be randomised (allocation ratio 1:1) to a strategy of either fresh embryo transfer or thawed frozen embryo transfer (typically four to six weeks later and usually within three months of egg collection).

Randomisation will be undertaken by the research nurse or a delegated member of the research team using a secure web-based centralised system (with 24/7 telephone backup 365 days/year) hosted by the National Perinatal Epidemiology Unit Clinical Trials Unit (NPEU CTU), University of Oxford. The randomisation will employ a minimisation algorithm to balance across the following factors: fertility clinic, woman’s age (at the time of start of treatment i.e. ovarian stimulation), primary/secondary infertility, self-reported duration of infertility, method of insemination (IVF/ICSI or a combination of both) and number of previous egg collections (cycles).

### Treatment allocation

Blinding of the allocated intervention is not possible in this trial because of the nature of the treatments and statutory requirements of the regulatory body – the HFEA. Discussion with the couple about the time and day of embryo transfer is routinely conducted over the telephone on the third day after fertilisation of eggs in the laboratory in all IVF clinics as part of routine care. A member of the IVF laboratory (embryology) team or research delegate will inform consented eligible couples on day three of the outcome of randomisation over the telephone.

The process will be as follows:

- Standard care arm: Women will undergo fresh embryo transfer at the cleavage or blastocyst stage according to local protocols.
- Intervention arm: All good quality embryos will be frozen according to local protocols. Women will be contacted by their research nurse after randomisation and arrangements will be made for thawed frozen embryo transfer (typically this takes place within 4 to 6 weeks and usually within three months of the egg retrieval process). This will involve a few visits to hospital to prepare the endometrium.

### Withdrawal procedures

Couples will be able to withdraw their consent to take part in the trial at any time without giving a reason. Withdrawal from the intervention/study will not affect their ongoing care. Non-adherence to the allocated intervention may also occur; this is defined as a difference between the treatment allocation provided at randomisation and the allocation received by the woman at the time of embryo transfer. Non-adherence to the allocated intervention may occur if the clinician feels it is in the couple’s best interests, e.g. freezing all created embryos is necessary for medical reasons, or transferring fresh embryos for clinical reasons. In the case of a non-adherence to the allocated intervention, the couple, with their on-going consent, would continue to be part of the trial, with outcome data collected in the routine manner.

### Safety reporting

Foreseeable serious adverse events (SAEs) are those events which are expected in the patient population or as a result of the routine care/treatment of a patient. Foreseeable SAEs will be collected on the electronic Case Report Form (eCRF) as part of routine data collection. The following events are foreseeable in women or couples undergoing IVF treatment and as such do not require reporting as SAEs.

Foreseeable events relating to the female partner, or couple:

- Ovarian hyperstimulation syndrome (OHSS)
- Miscarriage
- Hypertensive disorders of pregnancy
- Antepartum haemorrhage
- Gestational diabetes mellitus (GDM)
- Multiple pregnancy
- Failure of any embryos to survive thawing

Foreseeable events relating to the baby, when born:

- Low birth weight
- Very low birth weight
- Large for gestational age
- Preterm delivery
- Very preterm delivery
- Small for gestational age

### Unforeseeable serious adverse events

An unforeseeable SAE is any event that meets the definition of a SAE and is not detailed in the list above as foreseeable. The following unforeseeable SAEs must be reported:

- Maternal death
- Stillbirth
- Congenital anomaly detected antenatally or postnatally
- Neonatal death

Unforeseeable SAEs will be reported up to six weeks post-delivery.

### Data collection

Data for both clinical and economic outcomes will be collected using bespoke electronic case report forms (eCRFs) and entered directly into the trial’s OpenClinica electronic database by the IVF centre’s research staff. Data will be single-entered and at the point of entry the data will undergo a number of validation checks to verify the validity and completeness of the data captured.

### Sample size calculation

The proposed primary outcome for this trial is novel and is not currently reported by IVF clinics or national regulatory bodies. This means that a number of assumptions have been made in order to determine the expected event rate in the control arm (receiving current standard treatment), which may in turn result in a degree of imprecision in the estimate.

The data from the HFEA [8], which collects data on all IVF cycles from all clinics in the UK, show that 25% of all women undergoing one episode of IVF treatment involving a fresh embryo transfer have a live birth, and 20% have singleton live births. These figures are for women of all age groups, not necessarily for women fulfilling the inclusion criteria for this trial in terms of the number of good quality embryos in their IVF cycle. No data are available regarding the healthy baby rate (live singletons born between 37 and 42 weeks with appropriate weight for gestation), the primary outcome for this study. For our trial population we anticipate that the control arm event rate is likely to be less than 25%, possibly as low as 17%. Although the live birth rate is expected to be higher in women with at least three good quality embryos (likely to have a better prognosis), we anticipate that the healthy baby rate in our trial population will be towards the lower end of the confidence interval, around 25%, taking into account the higher risk of preterm delivery and small for gestational age babies following IVF [9].

#### The following assumptions have been made for the sample size calculation

We have assumed a healthy baby rate of between 17% and 25% in women eligible for the trial (age under 42 years with three good quality embryos) undergoing standard care (fresh embryo transfer). Taking into account the extra time, effort and potential expense involved in freezing embryos and the delay in embryo transfer of up to three months, a panel of clinicians across the UK agreed that the strategy of freezing embryos would be considered clinically effective if the percentage of women having a healthy baby is increased by at least 8% in absolute terms. With 90% power and using a two-sided 5% level of statistical significance, we will need to randomise a total of 1086 couples (543 in each group) in order to be able to detect an absolute difference of 8% from 17% to 25% and 9% from 25% to 34% in the healthy baby rate, between fresh embryo transfer and transfer of thawed frozen embryos. The difference detectable differs slightly depending on the event rate in the standard care group, which will be reviewed periodically by the Data Monitoring Committee (DMC).

It is a regulatory requirement for clinics in the UK to report live birth outcomes (including number, weight, gestation and gender) to the HFEA after all embryo transfers i.e. there will be no loss to follow-up. Therefore, we have not taken into account loss to follow-up for these sample size calculations. It is anticipated that a proportion of those consented may not reach randomisation (e.g. those not having three good quality day three embryos or requiring all embryos to be frozen for medical reasons); therefore a higher number will need to be consented.

### Analysis

A detailed Statistical Analysis Plan (SAP) will be developed and agreed by the Trial Steering Committee (TSC) before the analysis is undertaken. The analysis and presentation of results will follow the most up-to-date recommendations of the CONSORT group. Baseline demographic factors and clinical characteristics of the woman will be summarised with counts and percentages for categorical variables, means (with standard deviations) for normally distributed continuous variables, or medians (with interquartile ranges) for other continuous variables.

All outcomes will be analysed in the groups to which they are assigned, regardless of deviation from the protocol or treatment received under the intention-to-treat analysis principle. All comparative analyses will adjust for the minimisation factors wherever possible. Binary outcomes will be analysed using a log binomial regression model, or using a log Poisson regression model with a robust variance estimator if the binomial model fails to converge. Linear regression will be used for normally distributed continuous outcomes and quantile regression for skewed continuous outcomes. Comparative analyses will entail calculating the adjusted risk ratio (RR) and 95% confidence interval (CI) for the primary outcome, adjusted RRs and 99% CIs for all binary secondary outcomes, adjusted mean differences (with a 99% CI) for normally distributed continuous secondary outcomes, or median differences (99% CI) for skewed continuous secondary outcome variables (unless the data can be transformed to normality). For neonatal secondary outcomes (e.g. low birth weight, small for gestational age, congenital anomaly and perinatal mortality) the adjusted analysis will also account for the anticipated correlation in outcomes between multiple births.

### Pre-specified subgroup analysis

The consistency of the effect of electively freezing embryos followed by thawed frozen embryo transfer on the primary outcome across specific subgroups will be assessed using the statistical test for interaction. Pre-specified subgroup analyses are (i) woman’s age (test for trend), (ii) fertility clinic, (iii) cleavage vs blastocyst embryo transfer, (iv) single vs multiple embryo transfer, (v) number of previous embryo transfer cycles.

### Secondary analysis

The primary analysis for all primary and secondary outcomes will be by intention-to-treat. Secondary analyses will be performed to include the clinically relevant denominators as shown in Table 1. In addition, failure of embryos to survive after thawing (per embryo thawed) will be reported for the intervention group.

**Table 1:** Secondary analysis

Outcome	Clinically relevant Denominator
Miscarriage rate	per total number of women with a positive pregnancy test at two weeks +/− three days after embryo transfer
Multiple pregnancy	per total number of pregnant women with an ongoing pregnancy resulting in delivery
Gestational diabetes mellitus	per total number of pregnant women with an ongoing pregnancy resulting in delivery
Hypertensive disorders	per total number of pregnant women with an ongoing pregnancy resulting in delivery
Antepartum haemorrhage	per total number of pregnant women with an ongoing pregnancy resulting in delivery
Preterm delivery (< 37 completed weeks)	per total number of pregnant women with an ongoing pregnancy resulting in delivery
Very preterm delivery (< 32 completed weeks)	per total number of pregnant women with an ongoing pregnancy resulting in delivery
Low birth weight (< 2500 g at birth)	per total number of babies born
Very low birth weight (< 1500 g at birth)	per total number of babies born
High birth weight (> 4000 g at birth)	per total number of babies born
Large for gestational age (>90th centile)	per total number of babies born
Small for gestational age (<10th centile)	per total number of babies born
Congenital anomaly/birth defect	per total number of babies born
Perinatal mortality	per total number of babies born

### Economic evaluation

A formal economic evaluation will be undertaken to assess the cost effectiveness of the alternative approaches to treatment used in the trial. Resource use and costs will be estimated primarily from a health and personal social services perspective. However, personal time and travel costs, associated with any additional treatment-related visits which are not part of standard routine practice, will also be estimated via a short questionnaire administered at the time of embryo transfer. This is to be completed by both partners. In addition, longer-term social costs associated with child health outcomes will be modelled based on existing literature. Trial data collection instruments (eCRFs) will be used to capture participant level resource use associated with treatment, up to the trial end points of delivery or failure to become pregnant following the initial transfer. Appropriate unit costs [10, 11] will be used to value resource use events recorded in the case report forms. These costs will be summarised by treatment allocation group (by intention-to-treat), and presented in relation to the primary and secondary clinical outcomes. A cost-consequence balance sheet will be constructed to highlight the favoured strategy on cost and each clinical outcome at 12 months. The extra cost per additional healthy baby delivered (in the thawed frozen embryo transfer group versus fresh embryo transfer) will also be estimated using linear regression with adjustment for minimisation variables and baseline covariates as appropriate.

Many couples who fail to conceive following the initial embryo transfer will have access to subsequent frozen/thawed transfers, although the costs and outcomes associated with these will not be captured within the trial follow-up. Additionally, some adverse birth outcomes (e.g. preterm delivery, low birth weight) can have a far-reaching impact on costs and child health outcomes. Modelling will therefore be used to inform cost effectiveness over an extended time horizon. In order to do this, we will adapt an existing decision model [12] to simulate the progression of couples (who do not experience live birth following their initial embryo transfer) to the subsequent transfer of their remaining frozen embryos.

The model will also capture the longer-term cost and quality of life outcomes for any infants born as a result of treatment. The outputs of this modelling exercise will also be presented in the form of a cost-consequence balance sheet. Deterministic and probabilistic sensitivity analyses will be undertaken to characterise the uncertainty surrounding the estimated differences in costs and outcomes between approaches, and to assess the impact of changes in key model input parameters and assumptions.

### Study management and oversight arrangements

#### Trial Steering Committee (TSC)

The role of the TSC is to provide the overall supervision of the study. The TSC should monitor the progress of the study and conduct and advise on its scientific credibility. The TSC will consider and act, as appropriate, upon the recommendations of the Data Monitoring Committee (DMC) and ultimately carries the responsibility for deciding whether a trial needs to be stopped on grounds of safety or efficacy.

#### Data Monitoring Committee (DMC)

A DMC independent of the applicants and the TSC will review the progress of the trial at least annually and provide advice on the conduct of the trial to the TSC who will report to the Health Technology Assessment (HTA) programme manager. The committee will periodically review study progress and outcomes. The DMC will consist of an independent chair and at least two other independent members, who will be experts in their field, such as an embryologist, statistician or IVF clinician.

#### Project Management Group (PMG)

The study will be supervised on a day-to-day basis by the Project Management Group (PMG). This group reports to the TSC, which has overall responsibility for the conduct of the study. The PMG will meet regularly (at least monthly).

#### Trial Management

The trial co-ordinating centre will be at the NPEU CTU, University of Oxford, where the Trial Manager will be based. The NPEU CTU will be responsible for trial oversight, IT system/functions such as randomisation, clinical and administrative databases, all programming and statistical analyses, servicing both the DMC and TSC, and, in collaboration with the Chief Investigator and the Local Research Nurse for the general day-to-day running of the study including recruitment of sites and training of staff. A 24/7 (365 days a year) emergency helpline is available for out-of-hours queries relating to the trial. The economic analysis will be conducted at the University of Aberdeen.

#### Risk Assessment and Monitoring

A study risk assessment and monitoring plan has been completed as part of the development of this study by NPEU CTU. This risk assessment and monitoring plan will be reviewed at regular intervals during the course of the study to ensure that appropriate and proportionate monitoring activity is performed.

### Confidentiality, data protection and data management

Direct access to source data/documents (including hospital records/notes, clinical charts, laboratory reports, pharmacy records and test reports) will be granted to authorised representatives from the NPEU CTU, the Sponsor and host organisations to permit study related monitoring, audits and inspections.

Overall responsibility for ensuring that each participant’s information is kept confidential will lie with the study Sponsor who has delegated this responsibility to the NPEU CTU. All paper documents will be stored securely and kept in strict confidence in compliance with the Data Protection Act (2018). Data entered onto the eCRFs will be automatically transferred for storage in an electronic database hosted by NPEU CTU on behalf of the Sponsor, in which the participant will be identified only by a study specific number. The participant’s name and any other identifying details will be stored in a separate database also held by NPEU CTU on behalf of the Sponsor which will be linked to the database containing study data only by the participant’s study number. After the study has been completed and the reports published, the data will be archived. Electronic and paper documents will be archived by the NPEU using their secure archiving facilities, as detailed in NPEU Standard Operating Procedures.

Electronic files will be stored on a file server that has restricted access. The server is in a secure location and access is restricted to a few named individuals. Access to the building in which the NPEU CTU is situated is via an electronic tag and individual rooms are kept locked when unoccupied. Authorisation to access restricted areas of the NPEU CTU network is as described in the NPEU CTU security policy. Data will be processed on a workstation by authorised staff. The computer workstations access the network via a login name and password (changed regularly). No data are stored on individual workstations. Backing up is done automatically overnight to an offsite storage area. The location of the backup computer is in a separate department which has electronic tag access. Access to the room in which the backup machine is located is via a keypad system.

### Practical considerations

In order to participate in the trial, couples should have at least three good quality embryos on day three after egg collection. This is to ensure that they have enough embryos to survive the freezing/thawing process. The risk of failure of an embryo to survive thawing is minimal and the requirement to have at least three embryos should make the risk negligible. As there are no available data on the likelihood to achieve three good quality embryos, the impact on recruitment is unknown, however it is anticipated that at least half of those consented will not progress to take part in the trial.

There may be a long gap between consent and randomisation (Fig. 2). An individual’s medical and personal situation can change in between; hence there will be an expected drop out between consent and randomisation. This will be in addition to those who will not have three good quality embryos.

There will be no restriction of NHS or private sites. All couples will be entitled to participate, if they fulfil the inclusion criteria and they are having treatment in a clinic that is participating in the trial. There will be no extra cost to the patients participating in the trial even if they are funding their IVF treatment themselves.

Although this trial will only compare one fresh versus one thawed frozen embryo transfer, couples will be consented for follow-up. For those who consent to follow-up, we will report on cumulative live birth rate and long term outcomes of babies born (with appropriate approvals).

## Discussion

There is widespread interest in embryo freezing and a policy of freezing all embryos is increasingly being adopted globally. Although there are published trials, E-Freeze is specific to population and policies in the UK. This is a pragmatic trial; we expect that results will influence national guidance for practice to a wider group rather than a restricted group. To ensure this, IVF clinics continue with their normal processes of stimulation regimens, freezing protocols and embryo transfer policies. This will encourage more clinics to take part in the trial.

The aim of IVF is a healthy baby. This is the only trial with this outcome comparing fresh versus thawed frozen embryo transfer. The trial is also unique in exploring broader considerations, including both the emotional factors associated with the wait linked to embryo freezing and the cost both in terms of freezing processes and additional clinic visits for the couple. This will be the first trial in the world to explore and model the long term costs associated with a policy of freezing embryos and transferring later both from a healthcare and societal perspective.

## Abbreviations

CI: Confidence Interval
CIG: Co-Investigator Group
DMC: Data Monitoring Committee
eCRF: Electronic Case Report Form
GCP: Good Clinical Practice
GDM: Gestational Diabetes Mellitus
HFEA: Human Fertilisation and Embryology Authority
HTA: Health Technology Assessment
ICSI: Intracytoplasmic sperm injection
IVF: In vitro fertilisation
NICE: National Institute for Health and Care Excellence
NIHR: National Institute for Health Research
NPEU CTU: National Perinatal Epidemiology Unit Clinical Trials Unit
OHSS: Ovarian Hyperstimulation Syndrome
PMG: Project Management Group
REC: Research Ethics Committee
SAE: Serious Adverse Event
TSC: Trial Steering Committee
